# Association between the relative abundance of phyla actinobacteria, vitamin C consumption, and DNA methylation of genes linked to immune response pathways

**DOI:** 10.3389/fnut.2024.1373499

**Published:** 2024-04-04

**Authors:** Natália Yumi Noronha, Isabella Harumi Yonehara Noma, Rafael Fernandes Ferreira, Guilherme da Silva Rodrigues, Luzania dos Santos Martins, Lígia Moriguchi Watanabe, Marcela Augusta de Souza Pinhel, Isabelle Mello Schineider, Luísa Maria Diani, Daniela Carlos, Carla Barbosa Nonino

**Affiliations:** ^1^Department of Gynecology and Obstetrics, University Medical Center Groningen, Groningen, Netherlands; ^2^Faculty of Medicine of Ribeirão Preto, Department of Internal Medicine, University of São Paulo, Ribeirão Preto, Brazil; ^3^Department of Clinical and Toxicological Analyses, School of Pharmaceutical Sciences, University of São Paulo, São Paulo, Brazil; ^4^Department of Molecular Biology, Sao Jose do Rio Preto Medical School, São José do Rio Preto, Brazil; ^5^Faculty of Medicine of Ribeirao Preto, Department of Health Sciences, University of São Paulo, Ribeirão Preto, Brazil; ^6^Faculty of Medicine of Ribeirão Preto, Department of Biochemistry and Immunology, University of São Paulo, Ribeirão Preto, Brazil

**Keywords:** DNA methylation, Actinobacteria, vitamin C, immune response and nutrients, epigenetic

## Abstract

**Introduction:**

There is an emerging body of evidence that vitamin C consumption can modulate microbiota abundance and can also impact DNA methylation in the host, and this could be a link between diet, microbiota, and immune response. The objective of this study was to evaluate common CpG sites associated with both vitamin C and microbiota phyla abundance.

**Methods:**

Six healthy women participated in this cohort study. They were divided into two groups, according to the amount of vitamin C they ingested. Ingestion was evaluated using the 24-h recall method. The Illumina 450 k BeadChip was used to evaluate DNA methylation. Singular value decomposition analyses were used to evaluate the principal components of this dataset. Associations were evaluated using the differentially methylated position function from the Champ package for R Studio.

**Results and discussion:**

The group with higher vitamin C (HVC) ingestion also had a higher relative abundance of Actinobacteria. There was a positive correlation between those variables (*r* = 0.84, *p* = 0.01). The HVC group also had higher granulocytes, and regarding DNA methylation, there were 207 CpG sites commonly related to vitamin C ingestion and the relative abundance of Actinobacteria. From these sites, there were 13 sites hypomethylated and 103 hypermethylated. The hypomethylated targets involved the respective processes: immune function, glucose homeostasis, and general cellular metabolism. The hypermethylated sites were also enriched in immune function-related processes, and interestingly, more immune responses against pathogens were detected. These findings contribute to understanding the interaction between nutrients, microbiota, DNA methylation, and the immune response.

## Graphical abstract

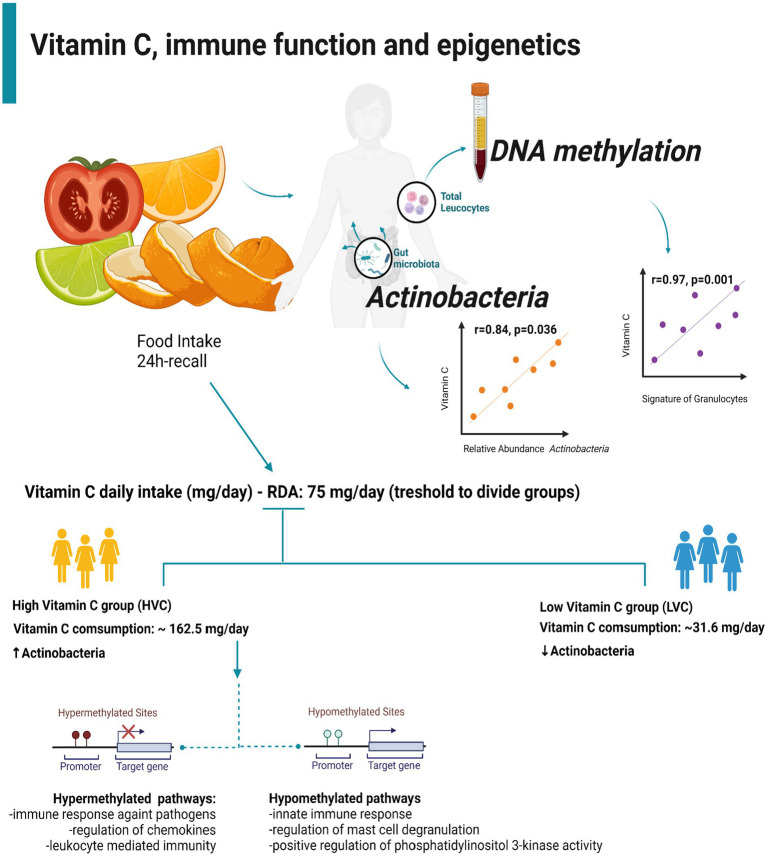

## 1 Introduction

In humans, the dominant phyla are *Bacteroidetes*, *Firmicutes*, *Actinobacteria*, *Proteobacteria, Fusobacteria*, and *Verrucomicrobia* (1). Nutrients influence the microbial community by promoting or inhibiting the growth of microorganisms, indirectly influencing the host’s metabolism and immune system (2). The gut microbiota synthesizes compounds as methyl and acetyl groups of vitamins. Consequently, it plays a role in DNA methylation and histone modifications, which influence physiological epigenetic changes. There is a growing body of evidence that nutrients can modulate the relative abundance of microbiota and that both nutrients and the diversity of bacteria in the microbiome can be related to DNA methylation (3). Especially vitamins can have an essential role in the microbiota’s abundance (4). Vitamin C is known for its antioxidant properties, and it is well-established that it can improve immune function. Little is known about the relationship of vitamin C with the bacterial phyla and the effect of this interaction on the epigenome. There are few studies in humans integrating epigenetic data, vitamin C, and microbiota relative abundance and how they are related. We hypothesize that vitamin C ingestion changed the microbiota, affecting DNA methylation. This study aimed to perform a pilot study to evaluate CpG sites related both to the relative abundance of specific bacterial phyla and vitamin C ingestion.

## 2 Materials and methods

### 2.1 Study design, ethics, and subjects

**Figure 1:**
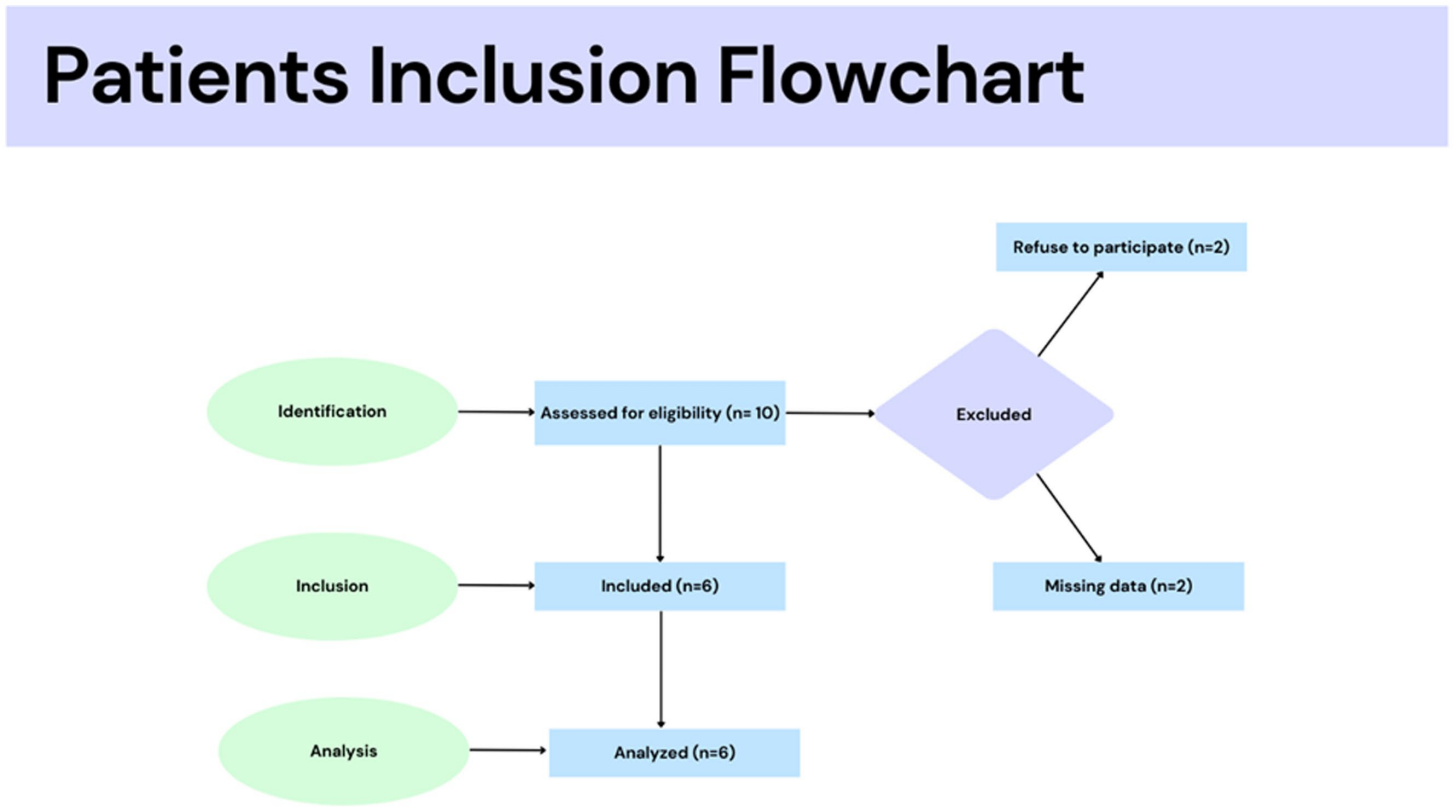
Participant flowchart included in this study.

This was a case–control pilot study. The Ethics Committee of Ribeirão Preto Medical School approved the study protocol at the University of São Paulo, Brazil (process CAAE: 57459816.1.0000.5440). Informed consent was obtained from all individual participants, and the inclusion criteria are shown in Figure 1. The study was conducted according to the Declaration of Helsinki. Six women, aged 30.8 ± 2.2, were subdivided into two groups (Figure 1).

### 2.2 Inclusion and exclusion criteria

Adult women without declared diseases were included in the study. Non-inclusion criteria included the use of antibacterial therapy, anti-inflammatory medicine, antifungal and anti-allergic medicines, probiotics and prebiotics, lactation, smoking, drinking, chemotherapy, a history of gastrointestinal surgery, gastrointestinal disease, diabetes mellitus, hypothyroidism or hyperthyroidism, the practice of physical exercise and dietary interventions already in course, and the use of new medical therapy that could influence the intestinal microbiota. The included patients were healthy and had no severe diseases diagnosed during the study.

The groups were divided based on the recommended reference values for vitamin C ingestion, resulting in three participants in each group. Data collection commenced on 19 November 2013, and concluded on 29 June 2015. During this period, each woman underwent the 24-h dietary recall three times. It is important to note that the 24-h recall method was used to assess the participants’ vitamin C consumption. This approach allowed us to evaluate their habitual vitamin C intake within the context of their daily dietary patterns. Blood collection and anthropometric measurements are only taken. Anthropometry, body composition, and resting metabolic rate were assessed following the protocol previously described by our group (5). Three 24-h dietary recalls were performed to evaluate the participants’ food consumption, and the software Dietwin® was used to calculate it. The subjects were evaluated after 12 h of fasting. Total blood was collected to assess glycemia, lipidogram, and gene expression analysis. Stool samples were collected to analyze the microbiota relative abundance.

### 2.3 Relative abundance of intestinal microbiota

DNA was extracted using the kit QIAamp® Fast DNA Stool Mini. The primer sequences used for each phylum were Firmicutes (F-ATGTGGTTTAATTCGAAGCA, R-AGCTGACGACAACCATGCAC), Bacteroidetes (F-GTTTAATTCGATGATACGCGAG, R-TTAASCCGACACCTCACGG), Actinobacteria (F-TGTAGCGGTGGAATGCGC, R-AATTAAGCCACATGCTCCGCT), Proteobacteria (F-CATGACGTTACCCGCAGAAGAAG, R-CTCTACGAGACTCAAGCTTGC), Verrucomicrobia (F-TCAKGTCAGTATGGCCCTTAT, R-CAGTTTTYAGGATTTCCTCCGCC), and rRNA 16S universal (F-AACAGGATTAGATACCCTGGTAG, R-GGTTCTTCGCGTTGCATC). The primers were at 10 μM. We used 20 ng of DNA in each well, ultra-pure water, and a master mix (SYBR Green, Promega®) with a final volume of 10 μL. After the reactions were performed, the results were analyzed using the Step One Plus® software (Thermo Fisher Scientific). We set the limit manually at 0.1. We use the 2^-ΔCt^ method to calculate the fold change and determine the relative abundance (6).

### 2.4 DNA extraction and methylation

DNA was extracted from whole peripheral blood using the Promega (Madison, Wisconsin, EUA) automated system with magnetic beads following the manufacturer’s instructions (7). Integrity was evaluated by agarose gel. Quality was evaluated using NanoDrop (Thermo Fisher, Waltham, Massachusetts, EUA). DNA from total leucocytes were firstly bisulfite converted using the EZ DNA Methylation Kit (Zymo Research Corporation, Irvine, CA, United States), then 500 ng were hybridized in the Infinium Human Methylation 450 k (IHM450K) BeadChip (Illumina, Inc., San Diego, CA, United States) (8). After the protocol, images were obtained using the iScan system.

### 2.5 DNA methylation data analysis and enrichment

The analysis was performed using R version 3.6.2 and ChAMP, a user-friendly R package available on Bioconductor. Raw intensity data from the IHM450K idats files were loaded on the R package, ChAMP (9). After the normalizations of the myNorm function, 409,819 probes continued in the pipeline for further processing, the missing data were removed, and the KNN imputation was performed (10). White blood cell type estimation was performed with the Houseman method (11). The intensities of the database were obtained from purified cells, and the IHM450K contains 473 CpG sites as a reference for the cell types. The obtained proportions were used to correct beta values using the Refbase function from the Champ package. Single value decomposition (SVD) was performed to check the principal components of the data variation. Limma package was used to determine differentially methylated positions (DMPs) (12), and a *p*-value of <0.05 and an FDR of <0.05 were considered significant. Linear regressions were performed to verify the association with clinical parameters (12). We performed Gene Functional Enrichment using the STRING database, we used as input the gene symbol from the mapped differentially methylated sites (13).

### 2.6 Gene expression validation of classic targets

The collected blood sample was used for RNA extraction to evaluate the gene expression of the target genes: *TNF-α*, *NFKB*, *IL-6*, *TLR4*, and *PI3kR1*. The phenol–chloroform method was used for RNA extraction. Reverse transcription (RT) was performed using the high-capacity kit (Applied Biosystems) to obtain cDNA. Quantitative PCR (qPCR) was performed using the TaqMan (Thermo Fisher Scientific) inventoried assays for the target genes. The results were evaluated by the 2-ddct method, using *ACTB* and *GAPDH* as endogenous genes and women without obesity as the normalizing group.

### 2.7 Statistical analysis

Shapiro–Wilk tests were used to verify normality, t-tests were used to verify differences between groups, and Spearman’s correlation was performed between vitamin C and phyla. For all analyses, we adopted a p-value of <0.05. Analysis was performed using the Statistica 7.0 software (StatSoft Statistica®).

## 3 Results

### 3.1 Sample

Six women (30.8 ± 2.2 years old) were included in this sample, and the group was divided by the recommendation for vitamin C ingestion (14). There were three women with high vitamin C (HVC) (30.7 ± 0.9 years old) and three with low vitamin C (LVC) (31.0 ± 2.9 years old) reference values. Table 1 shows all the collected clinical data. The two groups were similar for all evaluated parameters.

**Table 1:** Means and standard deviation of the anthropometric profile, body composition, biochemical parameters, and relative abundance of intestinal microbiota phyla.

Parameters	HVC (*n* = 3)	LVC (*n* = 3)	Reference value
Vitamin C (mg/day)	165.2 ± 23.6	31.6 ± 7.5*	^16^ 75 mg/day
Anthropometric profile			
Weight (kg)	62.8 ± 3.3	64.3 ± 4.2	
BMI (kg/m^2^)	22.4 ± 0.7	22.3 ± 0.4	^17^ 18.5–24.9 kg/m^2^
AC (cm)	72.3 ± 4.5	76.1 ± 2.5	^18^ 76.2 cm
Body composition			
FM (kg)	19.5 ± 1.6	18.2 ± 2.2	–
FM (%)	31.0 ± 1.0	28.3 ± 2.9	^19^ 28.8–35.7
FFM(kg)	43.3 ± 1.8	46.1 ± 3.6	–
FFM (%)	69.0 ± 1.0	71.7 ± 2.9	–
Biochemical Parameters			
Glycemia (mg/dL)	82.0 ± 7.3	77.0 ± 5.7	^20^ 70–99 mg/dL
CT(mg/dL)	159.7 ± 13.7	159.0 ± 22.6	^20^ 0–199 mg/dL
HDL-c (mg/dL)	63.8 ± 5.2	52.3 ± 15.5	^20^ 40–60 mg/dL
LDL-c (mg/dL)	73.7 ± 6.8	95.7 ± 13.8	^20^ 0–99 mg/dL
Relative abundance (2^-dct^)			
FC *Firmicutes*	24.8 ± 14.6	21.0 ± 13.8	–
FC *Actinobacteria*	6.0 ± 3.4	0.4 ± 0.1*	–
FC *Bacteroides*	20.5 ± 17.2	20.4 ± 15.0	–
FC *Proteobacteria*	0.1 ± 0.1	0.3 ± 0.3	–
FC *Verrucomicrobia*	0.4 ± 0.6	0.1 ± 0.0	–

Values are shown in mean ± standard deviation; BMI: body mass index; AC: abdominal circumference; FM: fat mass; FFM: free fat mass; CT: total cholesterol; HDL-c: high-density lipoprotein cholesterol, low-density lipoprotein cholesterol; FC: fold change (15). Recommended dietary allowance (RDA) of 75 mg/day for adult women (16), normal BMI range. (17). In the study of Rexrode et al. (18), abdominal circumference higher than 76.2 cm was associated with higher cardiovascular risk. According to Branco et al. (17), the % of body fat between 28.8 and 35.7 is considered normal in the Brazilian population. (19) Reference values biochemical tests Szwarcwald et al. (19). –: no reference value.

### 3.2 Phyla abundance and diversity

The HVC group had a higher relative abundance of Actinobacteria (Table 1). A strong positive correlation existed between the relative abundance of vitamin C and Actinobacteria (*r* = 0.84, *p* = 0.018). Table 1 shows the individual percentual relative abundance of the analyzed phyla. As expected, Bacteroides and Firmicutes were the most abundant groups in all women’s intestinal microbiota.

### 3.3 Epigenetic analysis

**Figure 2:**
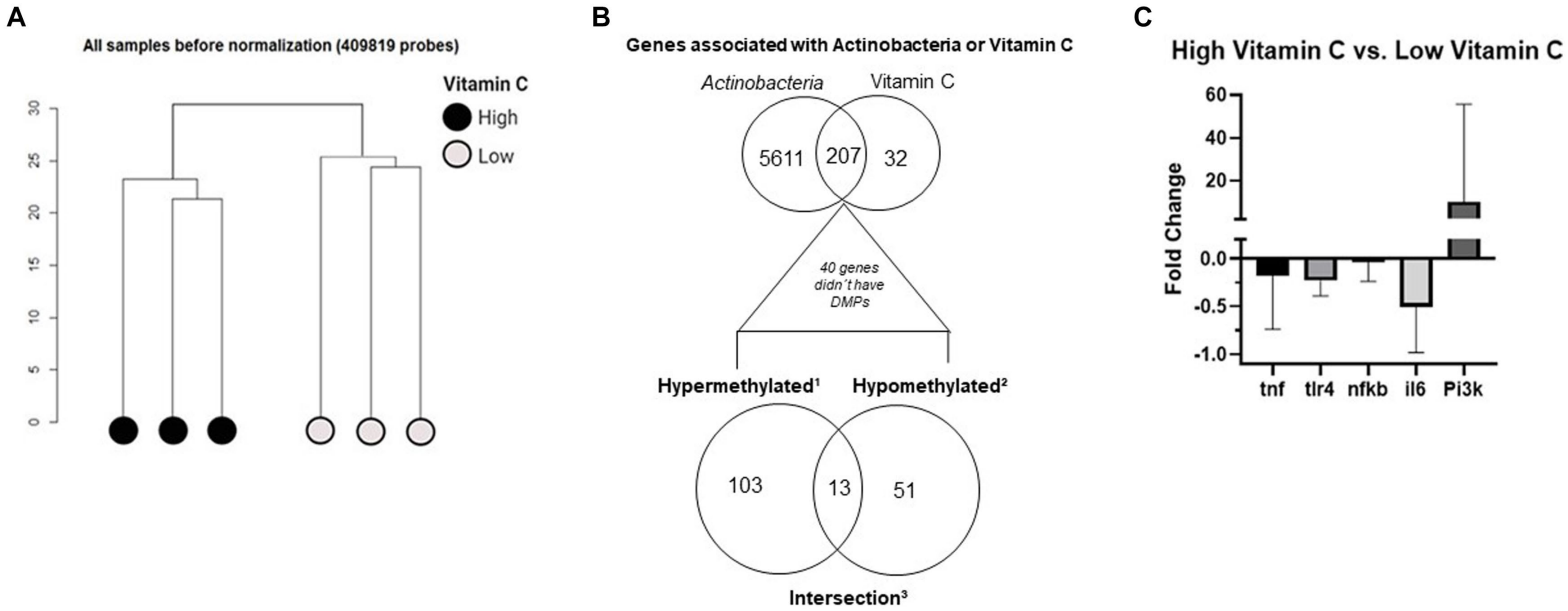
**(A)** Clusterization of raw DNA methylation data. **(B)** Venn diagram of common genes associated with vitamin C consumption and Actinobacteria relative abundance. ^1^Genes with only hypermethylated sites (*n* = 103, lower Venn diagram—left circle). ^2^Genes with only hypomethylated sites (*n* = 51, lower Venn diagram - right circle). ^3^Genes with both hypermethylated sites and hypomethylated sites (*n* = 13; lower Venn diagram—intersection). **(C)** Gene expression analysis of markers involved in the inflammation process: the different tones of gray represent each target analyzed, the bars are the fold change of the high vitamin C group compared to the LVC group, fold changes higher than 1 represent upregulation in the high vitamin C group, and negative fold changes represent downregulation in the high vitamin C group.

Regarding the quality control steps, the SVD analysis revealed that vitamin C was the most important variable in the metafile and better explained the data variation (*p* < 0.01). It was significant in the first principal component (PC1). Regarding bacteria relative abundance, only Actinobacteria were significant (*p* < 0.05) and influenced data variability. Figure 2A shows the clusterization of samples before normalization. The occurrence of the two clusters confirmed that the two groups had significant DNA methylation differences. The distance between samples was calculated based on the identified principal components previously obtained by SVD analysis. The generated outputs are part of a series of quality control analyses in the Champ pipeline. The analysis of QC parameters revealed that all samples were suitable for downstream analysis.

The association analysis retrieved that the associated sites were located in 239 genes for vitamin C and 5,818 genes for Actinobacteria; from those, there were 207 common genes (Figure 2B, upper Venn diagram). There were 40 genes that did not show any DMPs and are not shown in the subsequent Venn diagram (Figure 2B, lower Venn diagram). These 207 genes were classified into three categories according to their methylation status (Figure 2B, lower Venn diagram).

1. Genes with only hypermethylated sites (*n* = 103, lower Venn diagram - left circle).
2. Genes with only hypomethylated sites (*n* = 51, lower Venn diagram - right circle).
3. Genes with both hypermethylated sites and hypomethylated sites (*n* = 13; lower Venn diagram - intersection).

Based on the functional enrichment findings, we performed gene expression validation on some targets of interest (Figure 2C). We observed that the HVC group had, as expected, lower expression of the classical inflammation targets: *TNF-ɑ*, *TLR4*, *NFKB*, and *IL-6* (GO:0070944, GO:0050829, GO:0002443) and higher expression of the *PI3K* target (enriched in the pathways GO:0043552 and GO:0014068).

The common genes (*n* = 167) that had differentially methylated CpGs were functionally enriched. The analysis was performed separately for the genes that had hypomethylated and hypermethylated sites (Tables 2, 3). The genes that had hypomethylated CpGs had targets involved in the respective processes: immune function, glucose homeostasis, and general cellular metabolism. The hypermethylated sites were also enriched in immune function-related processes, and interestingly, more immune responses against pathogens were detected.

**Table 2:** Functional enrichment of the hypomethylated sites in the promoter region.

	Term description	False discovery rate
GO:0048014	Tie signaling pathway	0.0081
GO:0046069	cGMP catabolic process	0.0113
GO:0002040	sprouting angiogenesis	0.0257
GO:0002092	positive regulation of receptor internalization	0.0257
GO:0002376	immune system process	0.0257
GO:0006878	cellular copper ion homeostasis	0.0257
GO:0007154	cell communication	0.0257
GO:0007626	locomotory behavior	0.0257
GO:0008542	visual learning	0.0257
GO:0023052	signaling	0.0257
GO:0030097	hemopoiesis	0.0257
GO:0043114	regulation of vascular permeability	0.0257
GO:0043304	regulation of mast cell degranulation	0.0257
GO:0043552	positive regulation of phosphatidylinositol 3-kinase activity	0.0257
GO:0046068	cGMP metabolic process	0.0257
GO:0050918	positive chemotaxis	0.0257
GO:0051897	positive regulation of protein kinase B signaling	0.0257
GO:0060627	regulation of vesicle-mediated transport	0.0257
GO:0070374	positive regulation of ERK1 and ERK2 cascade	0.0257
GO:0072012	glomerulus vasculature development	0.0257
GO:1900181	negative regulation of protein localization to the nucleus	0.0257
GO:1901215	negative regulation of neuron death	0.0257
GO:2000251	positive regulation of actin cytoskeleton reorganization	0.0257
GO:2000352	negative regulation of the endothelial cell apoptotic process	0.0257
GO:0014068	positive regulation of phosphatidylinositol 3-kinase signaling	0.0332
GO:1904589	regulation of protein import	0.0358
GO:0001505	regulation of neurotransmitter levels	0.0406
GO:0045087	innate immune response	0.0445

**Table 3:** Functional enrichment of the hypermethylated sites in the promoter region.

	Term description	False discovery rate
GO:0046903	Secretion	0.0118
GO:0070944	Neutrophil-mediated killing of bacterium	0.0118
GO:0045073	Regulation of the chemokine biosynthetic process	0.0202
GO:0032940	Secretion by cell	0.0220
GO:0044130	Negative regulation of the growth of symbionts in the host	0.0220
GO:0050829	Defense response to Gram-negative bacterium	0.0238
GO:0045055	Regulated exocytosis	0.0254
GO:0042108	Positive regulation of cytokine biosynthetic process	0.0360
GO:0002443	Leukocyte-mediated immunity	0.0422
GO:0050776	Regulation of immune response	0.0491

**Figure 3:**
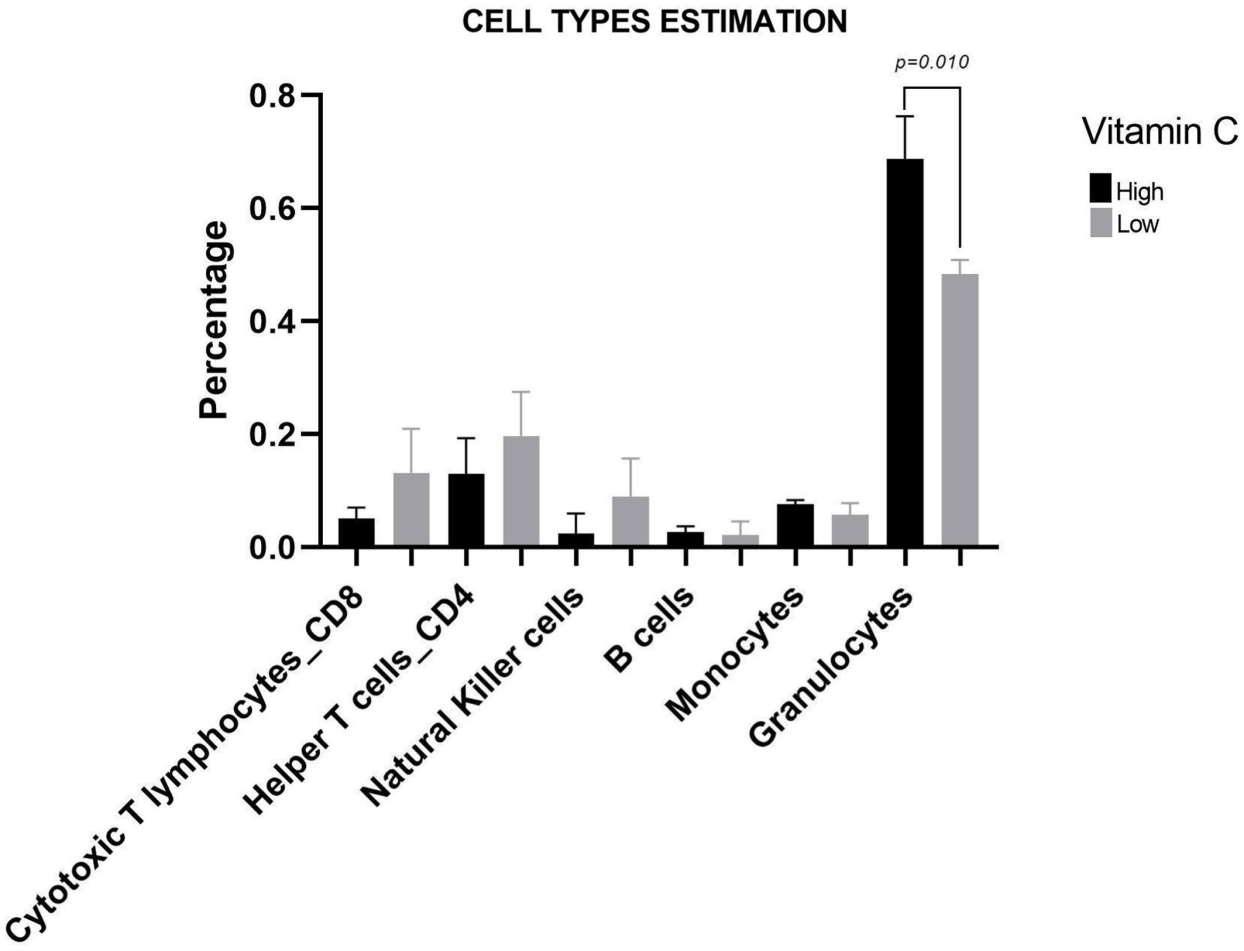
Cell type estimation graph based on the Houseman method. Results are presented as the mean, and the bars represent the standard deviation. *p*: *p*-value.

Regarding cell fraction estimation, the group with HVC consumption had 30% more granulocytes (HVC: 0.68 ± 0.06; LVC:0.48 ± 0.02; *p* = 0.0100) (Figure 3). Furthermore, vitamin C and granulocytes had a strong positive correlation (*r* = 0.97, *p* = 0.001). Vitamin C and the relative abundance of Actinobacteria were also positively correlated (*r* = 0.84, *p* = 0.036).

## 4 Discussion

Our results showed a positive correlation between the relative abundance of Actinobacteria and the consumption of vitamin C. The same relationship was described by Xu et al. (20), who reported the same association in piglets. Vitamin C, known as ascorbic acid, is a potent antioxidant, helping to protect cells from the damage caused by free radicals, and a cofactor for a family of biosynthetic enzymes and gene regulators. It contributes to supporting various cellular functions of the innate and adaptive immune systems. The role of ascorbate as a critical mediator by participating in epigenetic demethylation, as well as any genetic and environmental factors, such as gut microbiota, that influence ascorbate synthesis, absorption, transportation, and metabolism, could have significant consequences for the genome by modulating epigenetic control and consequences in health to control diseases (21). In addition, vitamin C serves as an essential cofactor for several monooxygenases and dioxygenases, including the 10–11 translocation (TET) family of enzymes, which participate in the process of DNA demethylation (22). Actinobacteria are Gram-positive and include three main anaerobe families (Bifidobacteria, Propionibacteria, and Corynebacteria) and one aerobe family (Streptomyces), with Bifidobacteria being the most common in the human gut. The Actinobacteria phylum represents a minority group of commensal bacteria but plays a role in the development and maintenance of gut homeostasis. Although it has been largely demonstrated that diet strongly influences gut microbiota composition, the relationship between diet and Actinobacteria is still unclear (23). Actinobacteria also modulate immune-inflammatory responses by inducing regulatory T-cells, and an unbalanced abundance has been observed in several pathological conditions (23). Indeed, there is growing evidence that bacterial phyla can exert a role in epigenetic regulation (3) in the pathways of the immune system and cell signaling.

Another study found an increase in Actinobacteria and Firmicutes after the administration of vitamin D (4), suggesting that the administration of vitamin D can modulate intestinal bacterial composition and increase the abundance of potentially beneficial bacteria. Among the DNA methylation sites that were commonly associated with Actinobacteria and vitamin C consumption, it was observed that the immune response was the most important biological process in this dataset. The hypomethylation in the promoter regions in individuals with higher consumption of vitamin C may alter the pathways of the immune system process. Interestingly, the pathways of functional enrichment from hypermethylated genes are involved in the negative regulation of symbiont growth in the host, and neutrophil-mediated killing of bacteria. This aligns with the observation that the group with high vitamin C may modulate an abundance of Actinobacteria essential for maintaining a healthy gut microbiota and a balanced immune system.

Inhibition of DNA and histone methylation further promotes the effect of ascorbate on T-cell differentiation, which suggests a role for ascorbate in the active demethylation of DNA and histone in T-cell maturation (23). As in our results, the group with higher ingestion of vitamin C had pathways to immune function, signaling, and hypomethylation of degranulation. It was also reported to be involved in the differentiation of Th1 and Th17, as well as cytokine gene expression and CD8+ T cell regulation (24). HVC levels had a lower expression of *TNF-α*, *TLR4*, *NFKB,* and *IL-6* markers. According to a review that showed vitamin C in peripheral blood, monocytes decreased the generation of the pro-inflammatory cytokines *TNF-α* and *IL-6*. Furthermore, vitamin C has been shown to attenuate *NFKB* activation in neutrophils. Cytokines are related to pro- and anti-inflammatory responses, and vitamin C can modulate this complex (25). Toll-like receptor 4 (TLR4) is a pro-inflammatory group characterized by AP-1 and NFKB activation. Immediate response by the innate immune system requires class *PI3K* activation (26, 27), which aligns with our results due to the increase in PI3K and the activation of the innate immune response in the HVC group. The PI3K/AKT signaling pathway regulates glucose metabolism, facilitates glycolysis, and inhibits gluconeogenesis. This pathway directly influences glucose metabolism by phosphorylating various metabolic enzymes and transport regulators (28). In the present study, the gene expression of this target and the functional enrichment retrieved two GO terms related to the PI3K pathway; however, the literature does not have enough publications to discuss this finding.

We found a positive correlation between vitamin C and granulocytes. It has already been shown that a high-ascorbate diet increases circulating granulocyte numbers in peripheral blood (29). Granulocytes have a role in secondary infections and in the host defense mechanisms of polymicrobial infection (30). Therefore, vitamin C may change the immune response by increasing granulocytes and improving the response against secondary infections.

The limitations of this study are about sample size and no serology test was performed to measure vitamin C. The 24-h recall was used for the daily consumption analysis of vitamin C. This tool is often neglected in research. However, it is widely used in clinical practice. Therefore, it is possible to assess the intake of micronutrients and macronutrients through 24-h recall, an essential database tool. In conclusion, modulating the microbiota through the consumption of vitamin C can alter methylation patterns responsible for immune responses. Those who ingest more vitamin C seem to have hypermethylated genes related to the innate immune response.

## Data availability statement

The datasets presented in this study can be found in online repositories. The names of the repository/repositories and accession number(s) can be found at: https://www.ncbi.nlm.nih.gov/, GSE254646.

## Ethics statement

The studies involving humans were approved by the Ethics Committee of Ribeirão Preto Medical School at the University of São Paulo, Brazil (process CAAE: 14275319.7.0000.5440). The studies were conducted in accordance with the local legislation and institutional requirements. The participants provided their written informed consent to participate in this study.

## Author contributions

NN: Conceptualization, Data curation, Formal analysis, Investigation, Methodology, Project administration, Writing – original draft, Writing – review & editing. IN: Conceptualization, Formal analysis, Investigation, Methodology, Writing – original draft, Writing – review & editing. RF: Conceptualization, Formal analysis, Methodology, Writing – original draft, Writing – review & editing. GR: Conceptualization, Formal analysis, Investigation, Writing – original draft, Writing – review & editing. LM: Conceptualization, Methodology, Supervision, Writing – original draft, Writing – review & editing. LW: Conceptualization, Data curation, Investigation, Supervision, Writing – original draft, Writing – review & editing. MP: Conceptualization, Methodology, Writing – original draft, Writing – review & editing. IM: Conceptualization, Methodology, Writing – original draft, Writing – review & editing LD: Data curation, Methodology, Writing – review & editing. DC: Funding acquisition, Project administration, Resources, Writing – original draft, Writing – review & editing. CN: Funding acquisition, Project administration, Resources, Supervision, Writing – original draft, Writing – review & editing.
